# A Kinetic Analysis of the Thermal Degradation Behaviours of Some Bio-Based Substrates

**DOI:** 10.3390/polym12081830

**Published:** 2020-08-15

**Authors:** Ananya Thomas, Khalid Moinuddin, Svetlana Tretsiakova-McNally, Paul Joseph

**Affiliations:** 1 Institute for Sustainable Industries and Liveable Cities, Victoria University, P.O. Box 14428, Melbourne, VIC 8001, Australia; khalid.moinuddin@vu.edu.au (K.M.); paul.joseph@vu.edu.au (P.J.); 2 Belfast School of Architecture and the Built Environment, Ulster University, Newtownabbey BT37 0QB, UK; s.tretsiakova-mcnally@ulster.ac.uk

**Keywords:** bio-based substrates, thermogravimetry tests, kinetic analysis, energy of activation, correlations

## Abstract

In the present paper, we report on a detailed study regarding the thermal degradation behaviours of some bio-sourced substrates. These were previously identified as the base materials in the formulations for fireproofing wood plaques through our investigations. The substrates included: β-cyclodextrin, dextran, potato starch, agar-agar, tamarind kernel powder and chitosan. For deducing the Arrhenius parameters from thermograms obtained through routine thermogravimetric analyses (TGA), we used the standard Flynn–Wall–Ozawa (FWO) method and employed an in-house developed proprietary software. In the former case, five different heating rates were used, whereas in the latter case, the data from one dynamic heating regime were utilized. Given that the FWO method is essentially based on a model-free approach that also makes use of multiple heating rates, it can be considered in the present context as superior to the one that is dependent on a single heating rate. It is also relevant to note here that the values of energy of activation (*E_a_*) obtained in each case should only be considered as apparent values at best. Furthermore, some useful, but limited, correlations were identified between the *E_a_* values and the relevant parameters obtained earlier by us from pyrolysis combustion flow calorimetry (PCFC).

## 1. Introduction

The worldwide interest in bio-based and degradable polymeric substrates has significantly accelerated in recent years [1,2,3,4]. Whilst bio-based materials are emerging as a suitable replacement to fossil fuel-based products, for obvious reasons, they also possess several undesirable properties that could significantly limit their applications [5,6,7]. For instance, most of these materials are thermally unstable compared to their synthetic counterparts, and are relatively flammable. These effects often exacerbate the limitations in their wider applicability [8,9]. In order to address such issues, it is imperative to study the thermal degradation profiles of biomaterials with a view to deciphering the physio–chemical processes underpinning their degradation behaviours and combustion attributes. Once this goal is achieved, it is prudent to seek ways of improving the thermal stability and means of mitigating the overall fire hazards of such materials. These attempts would definitely lead to the utilization of better performing systems as environmentally benign fireproof coatings for wood materials [9].

One of the most commonly used analytical techniques to study the thermal and thermo–oxidative degradation characteristics of polymeric materials is thermogravimetric analysis (TGA). It is also a common practice to derive useful kinetic parameters (i.e., the Arrhenius pre-exponential factor, *A*; the energy of activation, *E_a_*; the order of the reaction, *n*) from the thermograms obtained under different heating regimes [10,11,12]. One of the most useful empirical factors is the activation energy (*E_a_*) as it is directly related to the energetic needs for bond dissociation processes when polymeric chains undergo pyrolysis. In principle, the values obtained should also reflect the propensity of the individual bond cleavage process to occur when a material in question is progressively heated. This, in turn, can provide useful insights into the condensed-phase activity of the material while undergoing thermal cracking, and thereby its propensity to form combustible volatiles. Thus, once the useful information regarding the thermolytic profile of a material is gathered, the appropriate chemical modification(s) of the base matrix can be then designed in such a way so as to improve both its thermal stability and fire retardance [8].

Detailed kinetic analyses of thermograms are usually performed with a view to deducing the Arrhenius parameters. Generally, the available methods can be classified as belonging to non-isothermal or isothermal methods. In addition, several approaches within the two categories are also reported in the literature [13,14,15], among which the Flynn–Wall–Ozawa (FWO) method is the most prominent and is generally recognized as the most reliable one [16,17,18,19,20,21]. There are also several literature precedents that narrate techniques based on assumed models, which only use a single heating rate [20,22]. In the present work, we used the FWO method, as it is widely accepted as a model-free technique that can be utilized in determining the activation energies of materials using multiple heating rates [21,23]. In addition, we employed an in-house developed software, which utilized the data from a heating rate of 10 °C·min^−1^ [24,25]. The values of *E_a_* obtained from the two approaches were also compared in this study. Furthermore, we also sought some correlations between the *E_a_* values, obtained from the FWO method, and relevant tests parameters of the substrates that were gathered from pyrolysis combustion flow calorimetry (PCFC) [26,27,28,29].

## 2. Materials and Methods

For the current investigation, we employed six different substrates, such as β-cyclodextrin (MW = 1135), dextran (*M*_W_ ≈ 40,000), potato starch, agar-agar (Bacteriological No. 1), tamarind kernel powder and chitosan (medium molecular weight). All of these materials were obtained from the Aldrich Chemical Company, Melbourne, Australia, except for the tamarind kernel powder, which was also sourced locally from Melbourne, Australia. The substrates were used as received without further purification. The detailed structural features in each case are published elsewhere [8]. All of the materials were dried in a hot air oven (ca. 60 °C) for at least 16 h.

The thermogravimetric analyses (TGA) were performed on samples (ca. 5–10 mg, in the form of a powder) under an atmosphere of nitrogen, from 30 to 800 °C using a Mettler-Toledo instrument. The runs were also repeated at five heating rates (5, 10, 20, 30 and 60 °C·min^−1^). The reproducibility at each of the heating rates, and with different masses of each material, was also periodically checked by performing duplicate/triplicate runs. The primary aim of the TGA analyses was to obtain the Arrhenius parameters (*A* and *E_a_*). The relevant kinetic parameters were also deduced by employing a proprietary software that was developed in-house [24,25].

## 3. Results and Discussion

For the calculations involving the Flynn–Wall–Ozawa (FWO) method [16,17,21], initially, the data points obtained from the TGA runs at various heating rates were transferred into an Excel file, and subsequently, the degrees of conversion (i.e., the *α* values) were calculated using the following formula:

*α* = (*m_i_* − *m_t_*)/(*m_i_* − *m_f_*) (1)

where *m_i_* is the initial mass of the sample, *m_f_* the corresponding final mass, and *m_t_* the mass at a particular instance (i.e., time = *t*). After this, plots were constructed using the logarithm of the heating rates (i.e., log *β*) as the ordinate and a reciprocal of the temperature (1/*T*) corresponding to *α* value as the abscissa. As expected, the plots were linear, typically having an R^2^ value of ca. 0.93 (for example, in the case of tamarind, as given in Table 1 below).

The second method was based on a bespoke software that was primarily developed in-house [24,25]. In this approach, one of the non-isothermal thermograms was chosen. Here, as in all cases, we chose the thermogram obtained at a relatively low heating rate of 10 °C·min^−1^, as this is expected to capture the majority of the underlying steps in the thermal degradative pathway of the substrate in question (see also Figures 3–7 for an overlay of the thermograms).

### 3.1. Detailed Kinetic Analysis

Generally, the thermal and thermo–oxidative degradation of polymeric materials are complex processes involving consecutive and/or parallel steps. However, for the sake of simplicity, the kinetic analysis of the data from a TGA curve is often performed using a single step kinetic equation [13]. Furthermore, during the TGA runs, both isothermal and non-isothermal degradation regimes are adopted experimentally. It is also quite evident here that the isothermal approach is a thermodynamically more robust procedure than the latter one, where single or multiple heating rates are employed. In addition, it is also assumed that during the mathematical treatment of the data, the temporal integral (isothermal) is transformed to fit the multiple heating regime (non-isothermal), and that this is not going to affect the reaction kinetics. However, for a complex, multi-step process, this assumption may not be valid [22,30]. Therefore, this inherently limits the application of the relevant parameters, especially the values of *E_a_* that are computed from the non-isothermal methods [23]. Whilst these values are still useful, particularly to compare unmodified and modified polymeric systems, their validity in predicting the performance, or indeed the life cycle predictions, of a particular material, should be treated with caution [24,25]. As already mentioned, the values obtained in the present work, through the use of non-isothermal heating regimes, both single and multiple rates, can only, at best, be considered as apparent values of *E_a_*.

#### 3.1.1. Flynn–Wall–Ozawa (FWO) Method

**Figure 1:**
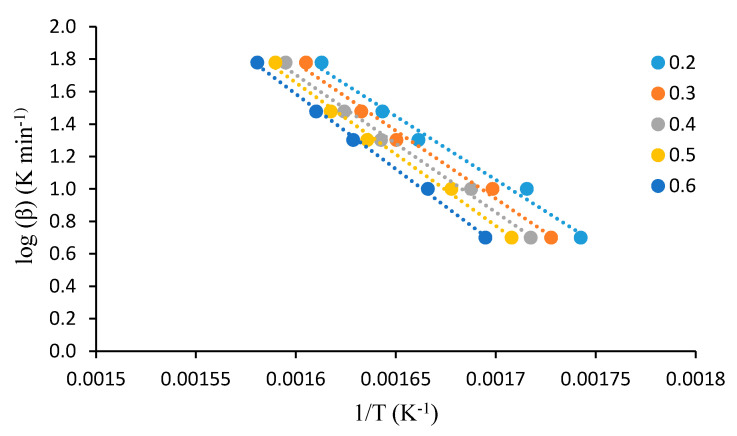
A plot of log *β* vs. 1/*T* at various *α* values (given as the inset) for β-cyclodextrin.

**Figure 2:**
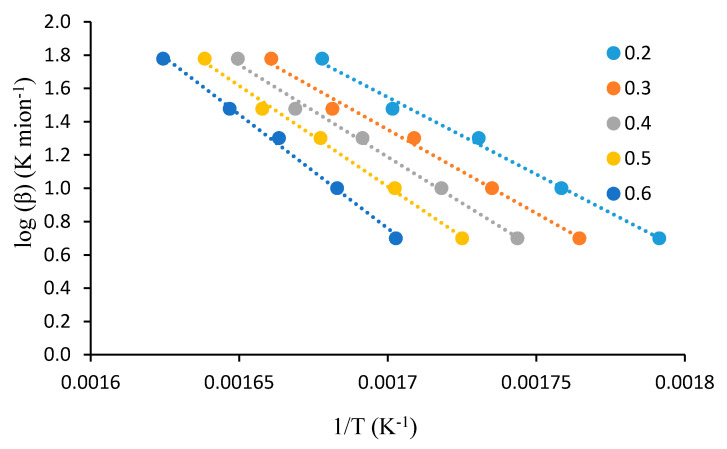
A plot of log *β* vs. 1/*T* at various *α* values (given as the inset) for dextran.

**Figure 3:**
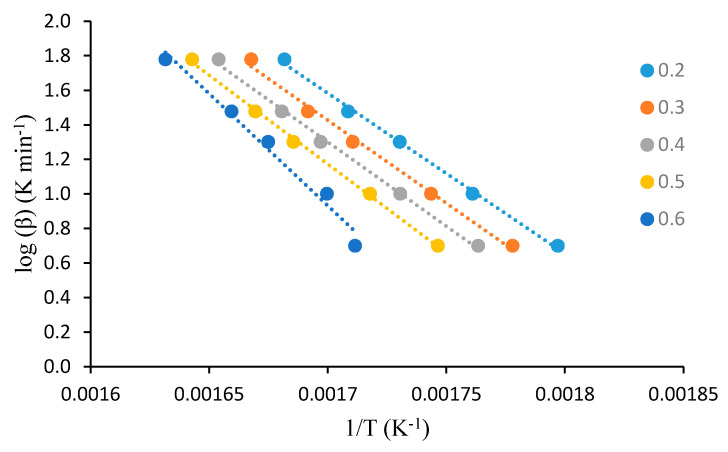
A plot of log *β* vs. 1/*T* at various *α* values (given as the inset) for potato starch.

**Figure 4:**
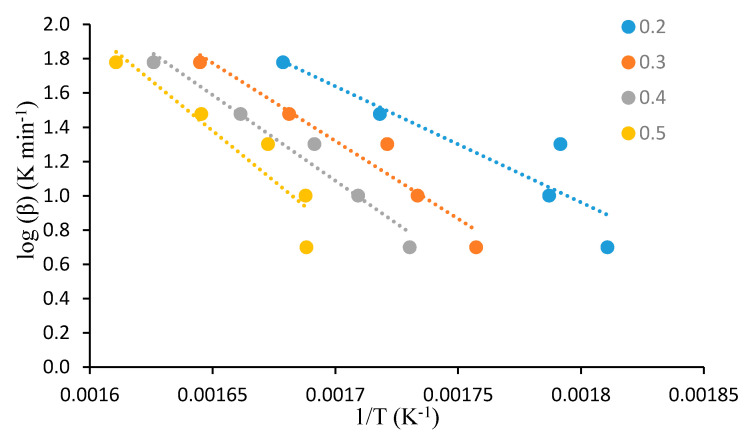
A plot of log *β* vs. 1/*T* at various *α* values (given as the inset) for tamarind.

**Figure 5:**
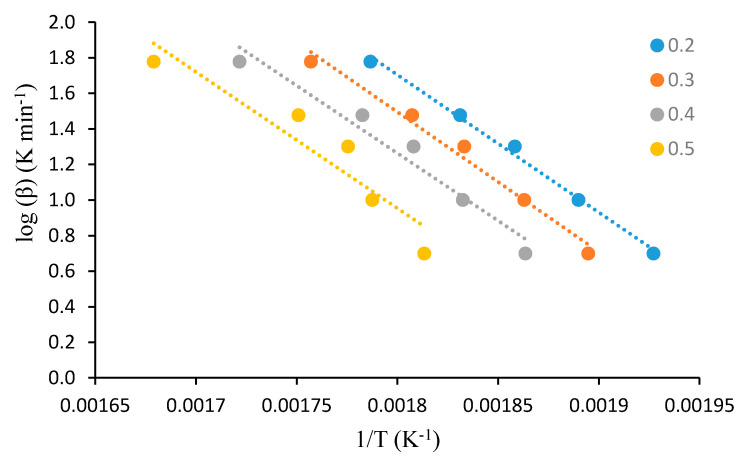
A plot of log *β* vs. 1/*T* at various *α* values (given as the inset) for agar-agar.

**Figure 6:**
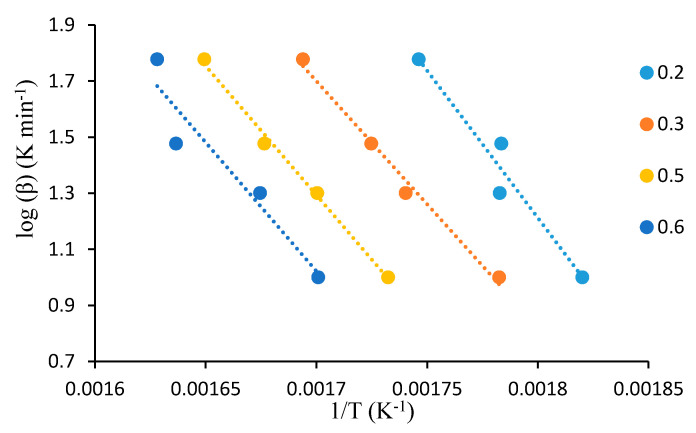
A plot of log *β* vs. 1/*T* at various *α* values (given as the inset) for chitosan.

For this analysis, the dynamic TGA analyses of the unmodified substrates, such as β-cyclodextrin, dextran, potato starch, agar-agar, tamarind kernel powder and chitosan, at various heating rates of 10, 20, 30, 40 and 60 °C·min^−1^, were carried out under an atmosphere of nitrogen. This method demonstrated that plotting log heating rate (*β*) against 1/*T_α_* generally gave straight lines with a slope equal to −0.4567(*E_a_/R*) (see Figure 1, Figure 2, Figure 3, Figure 4, Figure 5 and Figure 6). This is based on the following equation [7,8]:

log_10_*β* = −2.315 + log_10_(*AE_a_*/*R*) − log_10_*g*(*α*) − 0.4567(*E_a_*/*RT_α_*) (2)

However, in the case of carbohydrate substrates, it is worthy to note that the higher and lower values of *α* did not provide the expected linearity as envisaged classically by the Flynn–Wall–Ozawa method. For example, in the case of potato starch, the log *β* vs. 1/*T* plots only gave straight lines for *α* values, typically, between 0.2 and 0.6. At lower values of *α* (i.e., *α* < 0.2), the mass loss effects also include the elimination of physically bound water, which, in turn, do not require the energetic needs for breaking of covalent bonds. On the other hand, at higher values of *α* (i.e., *α* > 0.6), the primary/secondary oxidation of predominantly carbonaceous residues are bound to occur. In both instances, non-Arrhenius-type mass losses are highly likely, and therefore result in the observed deviations from linearity. This type of behaviour is not uncommon, especially in the case of lignocellulosic materials [31]. Essentially, we followed the same methodology in the case of the remaining substrates, which are given below.

The corresponding activation energies, in kJ·mol^−1^, for each of the values are tabulated below (Table 2).

#### 3.1.2. Method Using the Propriety Software

**Figure 7:**
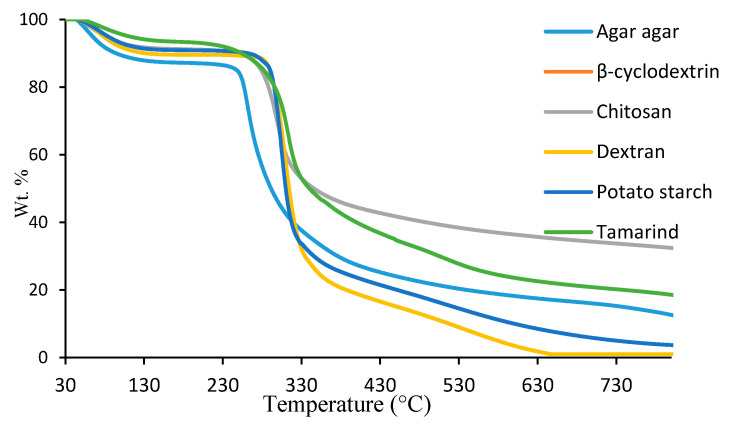
An overlay of thermogravimetric curves (TGA) at 10 °C·min^−1^ of all the six substrates.

The theoretical and computational approaches for this method are published elsewhere, in detail, by our research group [24,25]. The algorithms and associated software suite were devised in-house, to facilitate a convenient method of analysis for a wide range of non-isothermal TGA data. The overall approach delivers the so-called kinetic triplet (i.e., *A*, *E_a_* and *n*) information, and also enables one to assess whether the analysis has been appropriate in so far as the degradation occurred by a single mechanism over the temperature range. Taken together, the algorithms provide a seemingly useful approach to obtaining plausible kinetic triplets for a given system under investigation, provided that complexities, such as mechanistic changes are not encountered during the non-isothermal experiment (see Table 3). It should be noted here that, for the analyses using the method, we chose a moderate heating rate of 10 °C·min^−1^ as it is assumed that, at this heating rate, most of the representative degradation pathways of the substrates are essentially captured. An overlay of the corresponding thermograms is given below (Figure 7). As expected, all the substrates lost their moisture contents, followed by dehydration reactions, main chain transformation, forming different volatiles and finally resulting in varying amounts of char residues [8].

The results obtained from both methods are summarized in Table 4.

Given that the FWO method involves multiple heating rates, the values of *E_a_* obtained could be considered as more reliable than the output from the proprietary software, where the data points accrued through a single heating rate are used as the preliminary input parameters. Furthermore, the former method (i.e., the FWO method) is essentially a model-free option, whereas the latter method has the flexibility to choose from a host of possible models (from about 14 in total). However, the choice of the preferred model in the current work is based, primarily, on the nearest value of *E_a_* that corresponds to the value calculated through the FWO method. Here, it is also relevant to note that, in doing so, the corresponding R^2^ values were either 0.9, or above, indicating a strong correlation for the linear fit. In addition, the orders of the values for the Arrhenius factor were within what is normally expected for bond-cleavage processes; however, their absolute values may not bear any correlation with the actual physio–chemical processes that accompany such bond breaking reactions. In any case, the computed value and the correspondingly chosen values for *E_a_* should be only considered as apparent values that are useful in some instances for the purpose of comparison amongst closely related substrates. It is also relevant to note here that the *E_a_* values, calculated through the FWO method, for any given substrate showed variation with the corresponding α values, and the associated standard deviations also differed substantially, depending on the substrate in question (see in Table 2). Such variations could be attributed to the differences in the chemical nature and constitution of the different substrates.

#### 3.1.3. Correlation of *E_a_* Values with Some Relevant Combustion Parameters

We already reported on some of the relevant combustion parameters of the base substrates that were obtained through the pyrolysis combustion flow calorimetric technique (PCFC) [8]. These included: peak heat release rate (pHRR), total heat released (THR), heat release capacity (HRC), heat of combustion (*h_c_*) and char yield [26,27,28]. Through the present investigation, we also endeavoured to seek any correlations between these parameters and the values of the energy of activation. For this purpose, the table containing the values from PCFC measurements, reported previously [8], was reproduced (Table 5). Here, it is to be noted that the heating rate in the TGA and the heating rate during the PCFC were selected to have the same value (i.e., 60 °C·min^−1^ in TGA and 1 °C·s^−1^ in PCFC). However, owing to the inherent differences in the sensitivity/accuracy of the two types of instrumental techniques, there will be, invariably, some degree of deviance among empirical parameters.

In the table given below (Table 6), the values of *E_a_* (obtained from the FWO method), THR, *h_c_*, HRC and pHRR are given for the substrates with a view to identifying any trends in the data. As can be seen, the values of THR, *h_c_*, HRC and pHRR noticeably varied amongst the substrates. In the case of THR values, there is a smooth gradation with increasing values of *E_a_* for all the substrates, except in the case of chitosan (where the value recorded was the lowest; 6.60 kJ g^-1^). However, the trends, especially in other cases (i.e., for values of *h_c_*, HRC and pHRR), if at all present, were not smooth, and among the substrates, chitosan showed particularly lower values for THR and pHHR. β-cyclodextrin, starch and dextran showed similar variations for HRC and pHRR, whereas in all other cases, no discernible trends were observed. As the calculated values of *E_a_*, from the FWO method, essentially reflect the energetic needs for bond cleavage reactions, higher values are, therefore, expected to result in corresponding decreases in the values of some of the relevant combustion parameters (such as THR, HRC and pHRR, as in the present case). Furthermore, any deviations from a uniform gradation in the values could be attributed to the differences in the chemical nature and constitution among these substrates.

## 4. Conclusions

With a view to obtaining the Arrhenius parameters (primarily *A* and *E_a_*) of the base substrates, we used the well-known Flynn–Wall–Ozawa method, which employed five heating rates, and an in-house proprietary that utilized only one heating rate. Given that the FWO method is essentially based on a model-free approach that also makes use of multiple heating rates, it can be considered, in the present context, as superior to the in-house method, where the input data are essentially gathered from a thermogram obtained at a heating rate of 10 °C·min^−1^. Furthermore, the in-house method furnishes different values of *E_a_* depending on the model in question. In other words, for obtaining the activation energies of carbohydrate-based substrates, the FWO method seems to work more effectively than the in-house method, and hence we chose the *E_a_* values obtained through the FWO method for the correlation studies. However, when it comes to obtaining other kinetic parameters, including the *A* value, the in-house method gives a straightforward value, which would otherwise require tedious calculations (i.e., through the FWO method). In summary, we found both methods useful; however, the values of *E_a_* obtained in each case should only be considered, at best, as apparent values. Furthermore, we were also able to observe limited correlations between the energy of activation and some relevant parameters measured through the PCFC technique.

## Figures and Tables

**Table 1:** The values of abscissa (1/*T*) and ordinate (log *β*) for tamarind for the FWO method (at *α* = 0.2).

Sl. No.	log *β* (K·min^−1^)	Temp (°C)	Temp (*T*) (K)	1/*T* (K^−1^)
1	0.6989	279	552	0.00181079
2	1.0000	287	560	0.00178704
3	1.3010	285	558	0.00179163
4	1.4771	309	582	0.00171812
5	1.7782	322	595	0.00167868

**Table 2:** Activation energies (kJ·mol^−1^) of each substrate for different *α* values.

Sl. No.	*α*	Potato Starch	β-Cyclodextrin	Dextran	Agar-Agar	Tamarind	Chitosan
1	0.2	169	143	169	141	123	191
2	0.3	175	152	183	143	165	160
3	0.4	178	154	202	138	182	*
4	0.5	187	161	220	140	213	168
5	0.6	236	169	249	#	#	167
	**^†^ STDEV**	**27.1**	**9.78**	**31.4**	**2.08**	**37.5**	**13.5**

* The linear plot obtained when *α* = 0.4 turned out to be unreliable, as revealed by the corresponding value of *E_a_* < 50 units, which can be considered as incredibly low. # No linear fit of the data was obtained for tamarind or agar-agar (for *α* = 0.6). ^†^ Standard deviation for the values of the apparent activation energy for different substrates.

**Table 3:** Details regarding various outputs from the in-house method in the case of chitosan (as an example).

Sl. No.	Kinetic Model	Equation	*E_a_* (kJ·mol^−1^)	*A* (s^−1^)	R^2^
1	P1 Power Law	*α* ^1/*n*^	* -	-	-
2	E1 Exponential law	ln(*α*)	* -	-	-
3	A2 Avrami–Erofeev Model	[−ln(1 − *α*)]^1/2^	43.0	1.029 × 10^3^	0.9933
4	A3 Avrami–Erofeev Model	[−ln(1 − *α*)]^1/3^	26.0	1.880 × 10^1^	0.9931
5	A4 Avrami–Erofeev Model	[−ln(1 − *α*)]^1/4^	18.0	2.631 × 10^0^	0.9930
6	B1 Prout–Tompkins	[−ln(*α/*(1 − *α*))] + *C*	* -	-	-
7	R1 Contracting area	1 − (1 − *α*)^1/2^	86.0	1.682 × 10^7^	0.9944
8	R3 Contracting volume	1 − (1 − *α*)^1/3^	89.0	6.174 × 10^6^	0.9942
9	D1 One dimensional	*α* ^2^	164	8.417 × 10^13^	0.9952
10	D2 Two dimensional	(1 − *α*)ln(1 − *α*) + *α*	175	9.164 × 10^14^	0.9744
11	D3 Three dimensional	[1 − (1 − *α*)^1/3^]^2^	187	1.235 × 10^16^	0.9765
12	D4 Ginstling–Brounshtein	(1 − 2*α*/3) − (1 − *α*)^2/3^	179	2.181 × 10^15^	0.9751
13	F1 First order	−ln(1 − *α*)	95.0	2.330 × 10^7^	0.9880
14	F2 Second order	1/(1 − *α*)	32.0	1.487 × 10^1^	0.9050
15	F3 Third order	1/(1 − *α*)^2^	72.0	1.400 × 10^5^	0.7580

* No values for *E_a_* were given by the software, and the software yielded a zero value as the fitting factor.

**Table 4:** Relevant parameters obtained using the FWO and in-house methods.

Sl. No.	Substrate	*E_a_* (FWO Method (kJ·mol^−1^)	* *E_a_* (In-House Method) (kJ·mol^−1^)	*A* (s^−1^)	^#^ R^2^	Kinetic Model Chosen
1	*β*-cyclodextrin	156	118	7.74 × 10^9^	0.997	Avrami–Erofeev
2	Dextran	205	160	6.93 × 10^13^	0.993	First order
3	Potato starch	189	188	1.03 × 10^16^	0.976	Contracting volume
4	Agar-agar	141	140	4.78 × 10^11^	0.890	Two-Dimensional Diffusion
5	Tamarind	170	170	1.79 × 10^13^	0.990	Ginstling–Brounshtein
6	Chitosan	146	164	6.71 × 10^13^	0.995	One dimensional diffusion

* *E_a_* values were chosen in conformance with their corresponding values, obtained through Flynn–Wall–Ozawa method. ^#^ The R^2^ value denotes the linear fit parameter constructed through *g*(*α*) vs. *p*(*x*), where *p*(*x*) is deduced from an appropriate integral form of the Arrhenius equation [24,25].

**Table 5:** Relevant parameters from PCFC tests.

Sample	pHRR (W·g^−1^)	THR (kJ·g^−1^)	HRC (J·g^−1^·K^−1^)	Char Yield (wt. %)	* *h_c_* (kJ/g)
β-cyclodextrin	453	11.6	459	11.11	13.03
Dextran	289	10.4	288	^#^ -	^#^ 9
Potato Starch	363	10.4	368	12.50	11.84
Agar-agar	256	12.3	250	3.680	12.75
Tamarind	158	10.0	155	25.12	13.30
Chitosan	103	6.60	107	^#^ -	^#^ -

^#^ The value is not given here, as the pyrolysis char residue could not be determined accurately (the residue was rather sticky and blown-up in nature, hence, it was not possible to be retrieved fully after the run). * These values were calculated from the value of THR and the corresponding value of the pyrolysis residue [8].

**Table 6:** Energy of activation and some relevant parameters from PCFC tests.

Sl. No.	Sample	E_a_ (kJ mol^−1^)	THR (kJ g^−1^)	h_c_ (kJ g^−1^)	HRC (J g^−1^ K^−1^)	pHRR (W g^−1^)
1	β-cyclodextrin	156	11.6	13.03	459	453
2	Dextran	205	10.4	---	288	289
3	Potato Starch	189	10.4	11.81	368	363
4	Agar-agar	141	12.3	12.75	250	256
5	Tamarind	170	10.0	13.30	155	158
6	Chitosan	146	6.60	----	107	103
